# Impact of transition in work status and social participation on cognitive performance among elderly in India

**DOI:** 10.1186/s12877-019-1261-5

**Published:** 2019-09-11

**Authors:** Srei Chanda, Raman Mishra

**Affiliations:** International Institute for Population Sciences (IIPS), Mumbai, 400088 India

**Keywords:** Cognition, Retirement, Social participation, Depression, Elderly

## Abstract

**Introduction:**

Transition to the oldage marks a change in work and social participation. Socio-economic and physical conditions arising from this change pose a risk for cognitive outcomes among the elderly. Gender shows different pathways to deal with the pattern of participation and to maintain cognitive health. In India, work participation in the oldage is an outcome of financial deprivations and lack of support. At the same time, alterations in social interactions can induce stress and precipitate cognitive decline in oldage. A dearth of studies in this domain motivates us to estimate the effect of change in work and social participation on cognitive performance of the elderly in the Indian context.

**Methods:**

The study has used the cross-sectional data on 5212 elderly from the World Health Organization’s Study on global AGeing and adult health (Wave 1) (2007–08) in India. A composite score for cognition was generated. Interaction between gender, work status and social participation with respect to cognition was performed using multivariate linear regression. A linear prediction of the cognitive scores across all levels of social participation was post-estimated thereafter.

**Results:**

The study found that the elderly who were ‘presently working’ and showed ‘more’ social participation had a higher mean score for cognitive performance than their counterparts. Results of regression did not indicate any gender interaction with work or social participation. Participation in social activities ‘sometimes’ by those who were ‘retired’ or ‘presently working’ showed a positive and significant co-efficient with cognition among respondents. The post-estimated values for cognition specified that ‘retired’ and ‘presently working’ elderly had higher cognition scores. In the age group of 60–69 years, cognition scores were higher for those who were ‘retired’ and did ‘more’ social participation as compared to the other elderly.

**Conclusion:**

Cognitive aging is attenuated by higher participation in work and social activities. Adequate financial schemes or the pension system can protect the elderly from developing further stress. Retirement at an appropriate age, along with a reasonable amount of social participation, is a boon for cognitive wellbeing. Hence, building more support can contain the detrimental effect of participation restriction on cognitive outcome among elderly.

**Electronic supplementary material:**

The online version of this article (10.1186/s12877-019-1261-5) contains supplementary material, which is available to authorized users.

## Introduction

The transition from the adult age to older ages results in several inevitable life-course events. Among them, retirement from income-generating activities and reduced social participation pose a significant risk of cognitive loss in old age [1, 2]. Cognitive performance has long been studied to determine the threshold age of retirement, predominantly in the developed nations [3–5]. In a developing nation like India, greying of the population has an economic implication in terms of ascertaining the appropriate age for retirement [6–8]. The population of 60 years and above is projected to increase to 19% (2050) from 8% (2015). The increase in the population of the elderly (80 and above) is also high in India [9]. A substantial rise in aging has led to an increase in the incidence of physical limitations, cerebral pathologies, and alterations in socio-economic conditions like economic dependency, widowhood, and social isolation. There has also been an increase in the health burden due to mental morbidity and brain inactivity [10]. As an indicator of healthy aging, cognitive health in later life helps to understand the quality of life of the elderly [11].

Cognitive aging is explained by different stress pathways that consider intellectual engagement [2, 3, 12]. Retiring from employment-related activities adds worries in regard to financial security, health care, and health expenditure. The nature of cognitive decline after retirement varies with occupation, duration since retirement, and participation in alternative activities in later life as found by several longitudinal studies [2, 13–15]. Older persons with a higher cognition often continue to work until late in life. Early retirees tend to be individuals with a poor educational background and those who were engaged in low skill jobs [14]. However, in India, due to the absence of a strong social pension system or support from the family, individuals in older ages continue working out of compulsion [9]. Cognitive loss as a result of strenuous and involuntary work participation has been shown in a large number of studies, mainly in the advanced economies. The low- and middle-income countries too have started to face challenges arising from the growth of the longer living elderly.

Financial and social insecurity among the elderly heightens during the post-retirement phase due to an inadequate or dysfunctional social security system. Forced participation in employment in old age can be potent enough to alter the process of healthy aging among the fast-growing elderly population [16, 17]. Low-grade occupation is associated with poor educational attainment, which leads to a higher cognitive decline during old age [5]. Besides retirement from financial activities, reduced participation in social activities increases the risk of cognitive impairment in later life [2, 18, 19].

The theory of retirement and cognitive decline is supplemented by the idea of involvement in various social and leisure activities. Presence of a ‘we’ feeling, availability of social support, and social involvement influences cognitive health of the elderly. Group engagement is more important than individual engagement during old age to maintain cognitive wellbeing. At the extreme ages, group participation saves 10 years of cognitive outcomes if an individual remains active and involved [20]. In India, a number of older individuals are left alone in the family without adequate financial and social support. Increase in physical limitations, altered family structure, and low financial strength have been identified as stimulating agents for an increase in the feeling of insecurity among the elderly [19]. Effective social involvement and interaction are known to safeguard against the development of depressive symptoms. Low psychological resources negatively influence physical functions and cognitive wellbeing [21]. A better cognitive outcome can decrease the chances of degenerative diseases like dementia, depression, Alzheimer’s diseases, and other mental disorders in later years of life [7, 22]. Chronic and multi-morbid conditions are gradually increasing across the older ages, increasing the likelihood of functional limitations and disability [23, 24]. Among the elderly in India and low-middle income countries, a persistent increase in functional limitations has been observed as a result of multiple chronic morbidities [25]. It is well known that functional limitations through various ways aggravate psychological distress, which can further influence cognitive performance [26].

Cognitive performance is not the same across gender [11]. Gender shows a differential pattern in how work participation and social connectedness determine the cognitive behavior of individuals. In India, women have a lower participation in income-generating activities due to social norms and/or lower educational attainment. Physical inability restricts social participation and worsens the overall health conditions. Decline in functional health and the resultant comorbid conditions affect social engagement and increase mental stress significantly [27]. However, women benefit from close contact with others or from social participation. Through their participation in hobbies or volunteer groups, they preserve cognitive functions [28] that are otherwise at stake in the older ages.

In India, studies regarding cognitive functions among individuals are mostly limited to morbid conditions, education, and financial distress [11]. Even though a large number of elderly suffer from events like withdrawal from public interactions due to work restrictions/retirement, social isolation, discrimination, or social/financial neglect [9], the transitory impact of work and social participation across the age groups in later life is scarcely evaluated. Retirement affects the physical and mental health of the elderly; thus, restricting their participation in social activities. The importance and extent of social participation has rarely been understood in the Indian context, which is grappling with a rapid increase in the greying of population. The gender association of labor participation reveals a substantially higher male work participation and better pay for males than females, which is reflected in the differential financial security, decision making, and health outcomes in later life. Aging outcomes are being affected as the female elderly is living a longer life with poor health outcomes and support. The differential male and female patterns of social connectedness can demarcate the pathways of cognitive decline in older ages. The complex interactions of income-generating activities and social participation have been necessary to understand the implication of cognitive aging. Work and social participation restrictions together have the potential to significantly aggravate cognitive decline. In that context, there is a policy relevance to modify the labor norms, opportunities, and social development such that there is an inclusive development in India. This study focusses on the patterns of cognitive decline among the elderly through proximate and dynamic factors, those are, retirement and social connectedness. This is essential so as to understand the cognitive aging of the elderly as it is associated with health care and social support.

## Methods

### Data source and study population

For our study, we used the World Health Organization’s Study on global AGEing and adult health (WHO-SAGE, Wave1), conducted in India in 2007–08 to measure dimensions like health, living arrangement, care and support, and wellbeing among adults. It is a sister of the multi-country survey conducted in 6 countries, including India, China, Ghana, Russian Federation, South Africa, and Mexico. This is a nationally-representative sample survey conducted in 6 states of India, including Assam, Uttar Pradesh, West Bengal, Karnataka, Maharashtra, and Rajasthan (covering 37% of the population of India). A multistage clustered sample design and probability proportional to size (PPS) sampling method were used to conduct the survey. The states were selected on the basis of development indicators like infant mortality rate, female literacy, percentage of safe deliveries, and per capita income. A total of 5212 samples aged 50 and older were studied after dropping missing observations in selected cofounders. For the purpose of analysis, STATA 13 package was used in this study. The details of the study are mentioned in the WHO-SAGE (Wave 1) report [29].

### Independent variables

We selected the age group of 50 and above as representing the young elderly population that transitions to the age of retirement, which, for the most part, is 60 years in India. The age groups were categorized as: ‘50–59’, ‘60–69’, and ‘70+’. The place of residence was categorized as ‘rural’ and ‘urban’; gender as ‘male’ and ‘female’; marital status as ‘never married’, ‘currently married’, and ‘others’ (including widowed and separated); caste as ‘unreserved’ and ‘reserved’ (scheduled caste, scheduled tribe, other backward classes, and others); education as ‘no formal’, ‘primary’, ‘secondary’, and ‘college and more’; wealth index as ‘low’, ‘medium’, and ‘high’; and depression as ‘yes’ and ‘no’ [30].

Depression in the WHO-SAGE survey wave-1 was ascertained with a set of self-reported as well as symptom-based questions. The self-reported questions sought to determine whether any diagnosis had been done and if any medication or some other treatment had been taken during 12 months preceding the survey. The symptom-based questions sought to determine whether a respondent had experienced sadness, emptiness or depression/ loss of interest/ or decrease in energy lasting more than 2 weeks during 12 months preceding the survey. Respondents, who expressed agreement with any of the last three symptoms, were asked another set of symptom-based questions (Additional file 1). The survey questionnaire used for ascertaining depression is available at: http://apps.who.int/healthinfo/systems/surveydata/index.php/catalog/65

#### The working status

The working status variable was created as a combination of ‘ever worked’ and ‘currently working’ responses. Responses to ‘previously worked and not working presently’ generated the ‘retired’ variable. ‘Never worked’ was another category. It was out of scope to include the duration since retirement in our study.

#### Social participation

Social participation was assessed through nine indicators for how often an involvement in society had occurred during 12 months preceding the survey. The aspects were: public meeting, attending a club, participating in society or other meetings, etc. The responses were categorized as 1 for ‘daily’, 2 for ‘once or twice per week’, 3 for ‘once or twice per month’, 4 for ‘once or twice per year’, and 5 for ‘never’. A composite score was generated by using Multiple Classification Analysis (MCA) with the principal component. The response variable ‘social participation’ was categorized into three categories as 1 for ‘lesser’, 2 for ‘sometimes’ and 3 for ‘more’. The reliability score for the scale was 0.752 as measured through Cronbach’s alpha.

#### Functional restrictions

To assess the functional restrictions, the World Health Organization’s Disability Assessment Schedule (WHO-DAS) version-2 (a fuller set of activities inclusive of activity of daily living (ADL) and instrumental ADL (IADL)) was utilized in the study. The questions sought to ascertain the extent of difficulty experienced during 30 days prior to the survey in 22 activities, including ‘sitting for a long period’, ‘walking 100 meters’, ‘moving around inside home’, ‘getting to and using toilet’, ‘going somewhere with public or private transport’, etc. Response options were coded as 1 for ‘none’, 2 for ‘mild’, 3 for ‘moderate’, 4 for ‘severe, and 5 for ‘extreme/ cannot’. The response variable of functional restrictions was described as 1 for ‘low’, 2 for ‘medium’ and 3 for ‘high’. The Cronbach’s alpha for reliability was 0.948.

### Outcome variable

A battery of tests was performed in order to understand the cognitive functions of the individuals. These tests were: verbal fluency (VF), verbal recall (VF), and forward (FWD) and backward (BWD) digit span. To test the semantic memory (VF), respondents were asked to recall as many words (animal names) as possible in a one-minute time span. VR required recalling 10 words in 3 rounds of trial after listening to the words before each trial to saturate the learning curve. The test was inclusive of both immediate VR and delayed VR (performed after 10 min). For FWD and BWD, the individuals read a series of digits and were asked to recall the numbers in forward and backward directions. The test is used for measuring the working memory of an individual. For the purpose of comparison, we standardized the VR, VF, FWD and BWD scores. The summation of the 5 standard scores gave a composite value to assess cognitive performance.

### Statistical analysis

Descriptive statistics with percentages, mean, standard deviations (SD), and maximum and minimum score have been shown. To measure the impact of social connectedness on cognition, it was essential to observe the interaction between the two events (work and social participation) in the life of the older individuals. The effect of work and social participation on cognition was measured by using regression along with measuring the gender-specific effect on the type of participation. We applied a multivariate linear regression with the interaction among the prime variables in 3 different models to identify the significance of the predictor for gender and type of participation while controlling other background characteristics. Model 1 took into consideration the effect of individual background characteristics on the cognitive score of the respondents (full model). In model 2, the interaction of gender and work was observed after controlling other background characteristics. In model 3, we observed the effect of gender and social participation. In Model 4, the interaction of work and social participation was observed to measure the significance of the gender-neutral effect of participation on the cognition of the elderly after controlling other background characteristics. We checked the assumptions of the model – for e.g., linearity, normality, and multi-collinearity – before performing the analysis (Refer to Additional file 1). Post-estimation was used to measure the cognitive scores of the individuals with respect to their age groups and work participation scenario after performing the regression for Model 4. We kept social participation constant to understand the persistence of the social situations. This yielded 3 social participation groups as per the categories of the variable utilized for the study.

## Results

### Descriptive statistics for the study population

The description of the study population for the age groups of 50 and above together with the mean cognitive scores is presented in Table 1. In our study sample, the majority of the respondents were in the age group of 50–59 years (47.39%), were males (53.74%), resided in rural areas (73.81%), and were currently married (76.82%). Caste-wise, 63% of the respondents were unreserved and about 50% had no formal education. The study found that nearly 10% of the elderly were depressed. The mean cognitive score was seen to decline – from 0.794 to − 1.086 – as age increased. Females had lower mean cognitive scores (− 0.840) than males (1.045). Older persons living in urban areas had higher mean cognitive scores (1.347) than their rural counterparts (− 0.254). In terms of marital status, other categories (including widowed, separated, and divorced) had lower mean cognitive scores (− 1.326) than the currently (0.593) and never married (1.260) respondents. Unreserved castes scored higher on mean cognitive scores (0.537) than reserved castes (− 0.427). Higher levels of education were seen to be associated with better mean cognitive scores, the scores being as follows: no formal education (− 1.532), primary (0.631), secondary (2.525) and college (4.197). Similarly, better wealth conditions also improved the mean cognitive outcome of the elderly. Elderly in the low wealth category had a mean cognitive score of − 1.242, those in the medium category had a score of 0.046, and those in the high wealth category had a score of 1.594. The study found that 26.38% of the elderly had never worked, 29.45% had retired, and 44.17% were presently working. A higher cognitive outcome was exhibited by the elderly who were ‘presently working’ (0.852) than those who had ‘retired’ (− 0.032) and those who had ‘never worked’ (− 0.736). Elderly with a higher social participation showed a higher mean cognitive score. Those with ‘lesser’ social participation had a lower mean cognitive score (− 0.986) than those who participated ‘sometimes’ (− 0.141) and those who participated ‘more’ (1.412). The elderly suffering from depression comprised 10.28% of the study sample. Depression was linked with lower mean scores for cognition (− 0.528) than no depression (0.253). The elderly in the low functional restrictions category (measured by the WHODAS score) exhibited a higher mean cognitive score (1.406) than those in the medium (0.109) and high (− 1.148) functional restrictions categories. The mean cognitive score of the study sample (0.173), the SD (3.45), and the range (− 10.20 to 13.46) are shown in Table 1. Details of the descriptive statistics of cognitive scores are given in Additional file 1.

**Table 1:** Summary statistics by the background characteristics (Percentage, Mean, Standard Deviations (SD), Minimum and Maximum Cognitive Score of the respondents (*N*=5212).

Categories	Percentage	Mean	SD	Minimum	Maximum
Age Groups					
50–59	47.39	0.794	3.401	−9.858	12.803
60–69	33.71	0.005	3.326	−10.199	12.524
70+	18.90	−1.086	3.434	−9.593	13.463
Gender					
Male	53.74	1.045	3.318	−9.405	13.463
Female	46.26	−0.840	3.330	−10.199	11.762
Place of residence					
Urban	26.19	1.374	3.467	−9.858	12.391
Rural	73.81	−0.254	3.347	−10.199	13.463
Marital status					
Never married	0.94	1.260	4.125	−6.371	11.720
Currently married	76.82	0.593	3.364	−10.199	13.463
Others	22.24	−1.326	3.307	−9.858	10.365
Caste					
Unreserved	62.24	0.537	3.476	−9.858	13.463
Reserved	37.76	−0.427	3.332	−10.199	12.524
Completed education					
No formal	48.16	−1.532	2.913	−10.199	11.004
Primary	25.92	0.631	2.819	−9.310	10.192
Secondary	20.40	2.525	3.004	−8.833	13.463
College	5.53	4.197	2.910	−8.826	12.391
Wealth index					
Low		−1.242	3.065	−10.199	10.917
Medium		0.046	3.266	−9.858	11.564
High		1.594	3.407	−8.826	13.463
Work status					
Never worked	26.38	−0.736	3.341	−10.199	11.004
Retired	29.45	−0.032	3.589	−9.593	13.463
Presently working	44.17	0.852	3.282	−9.858	12.803
Social participation					
Lesser		−0.986	3.283	−9.858	11.004
Sometimes		−0.141	3.311	−10.199	11.176
More		1.412	3.325	−8.268	13.463
Depression					
No	89.72	0.253	3.466	−9.858	13.463
Yes	10.28	−0.528	3.263	−10.199	10.931
WHODAS score					
Low		1.406	3.420	−9.858	13.463
Medium		0.109	3.139	−9.027	10.501
High		−1.148	3.291	−10.199	11.004
Total		0.173	3.454	−10.199	13.463

### Association and interaction of work status and social participation on cognition

Table 2 gives an estimate of multivariate linear regression for the cognitive scores of the elderly with selected background characteristics. Model 1 predicted that having a higher age, being a female, residing in rural areas, and belonging to the reserved caste categories were significant and had a negative coefficient for cognitive performance among the elderly. Higher educational qualification, more wealth, and presence of depressive symptoms had a positive coefficient for cognitive outcomes. In terms of work participation, ‘retired’, and ‘presently working’ had a positive coefficient for the cognitive score. Higher social participation too showed a positive coefficient for the cognitive scores. Increase in functional restrictions led to higher WHODAS scores, resulting in a negative and significant coefficient for the cognitive score. The F-statistic for Model 1 was 166.3. In models 2 and 3, interaction of gender with work and social participation showed no significant outcome for the coefficient of cognition scores. However, the association between work and gender was positive. Model 3 yielded a negative direction of coefficient for the interaction between social participation and gender. In model 4, after controlling all other variables, we found the interaction between work and social participation to be negative and significant for selected interaction effects. As compared to those who had ‘never worked’ and had ‘lesser’ social participation, those who were ‘retired’ and participated ‘sometimes’ in social activities and those who were ‘presently working’ and participated ‘sometimes’ in social activities had a positive and significant coefficient for the cognitive outcomes. There was a stronger coefficient between being ‘retired’ and participating ‘sometimes’ in social activities (0.904) than between being ‘presently working’ and participating ‘sometimes’ in social activities (0.582). The F-statistics for Models 2, 3 and 4 were 150.4, 150.6 and 138.6, respectively. The interaction effect was significant for model 4 for the selected combinations. Model 4 also showed marginally higher R2 values (0.381) and a lower F-statistic than the full model, that is, Model 1 (R2: 0.378), which explains the strength of the model.

**Table 2:** Multivariate linear regression with selected background characteristics to predict the cognitive outcome of the elderly in India, 2007-08, in 4 models

Background variables	Model 1			Model 2			Model 3			Model 4		
	Coefficient	95%CI	*P* value	Coefficient	95%CI	*P* value	Coefficient	95%CI	*P* value	Coefficient	95%CI	*P* value
Age groups												
50-59	Ref			Ref			Ref					
60-69	−0.349	(−0.523;-0.175)	<.001	−0.350	(−0.525;-0.175)	<.001	−0.348	(−0.522;-0.173)	<.001	−0.353	(−0.522;-0.173)	<.001
70+	−1.018	(−1.247;-0.789)	<.001	−1.019	(−1.249;-0.790)	<.001	−1.022	(−1.251;-0.794)	<.001	−1.016	(−1.245;-0.788)	<.001
Gender												
Male	Ref			Ref			Ref					
Female	−0.202	(−0.416;-0.013)	0.065	−0.271	(−0.845;-0.303)	0.355	−0.066	(−0.398;0.265)	0.695	−0.189	(−0.403;-0.026)	0.084
Place of residence												
Urban	Ref			Ref			Ref					
Rural	−0.525	(−0.707;-0.343)	<.001	−0.525	(−0.708;-0.343)	<.001	−0.527	(−0.709;-0.345)	<.001	−0.524	(−0.706;-0.342)	<.001
Marital status												
Never married	Ref			Ref			Ref					
Currently married	−0.180	(−0.952;0.591)	0.647	−0.180	(−0.952;0.592)	0.647	−0.177	(−0.949;0.595)	0.652	−0.169	(−0.939;0.602)	0.668
Others	−0.580	(−1.368;0.209)	0.149	−0.579	(−1.368;0.209)	0.15	−0.577	(−1.366;0.211)	0.151	−0.556	(−1.344;0.231)	0.166
Caste												
Unreserved	Ref			Ref			Ref					
Reserved	−0.534	(-0.690;0.378)	<.001	−0.536	(−0.693;0.380)	<.001	−0.535	(−0.691;0.379)	<.001	−0.541	(−0.697;0.385)	<.001
Completed education												
No formal	Ref			Ref			Ref					
Primary	1.569	(1.375;1.762)	<.001	1.570	(1.376;1.764)	<.001	1.562	(1.369;1.756)	<.001	1.566	(1.373;1.759)	<.001
Secondary	2.718	(2.484;2.952)	<.001	2.719	(2.485;2.953)	<.001	2.717	(2.483;2.951)	<.001	2.722	(2.489;2.956)	<.001
College	3.819	(3.441;4.196)	<.001	3.819	(3.441;4.197)	<.001	3.823	(3.445;4.201)	<.001	3.836	(3.458;4.213)	<.001
Wealth index												
Low	Ref			Ref			Ref					
Medium	0.618	(0.430;0.807)	<.001	0.618	(0.430;0.807)	<.001	0.621	(0.433;0.810)	<.001	0.626	(0.438;0.814)	<.001
High	1.176	(0.967;1.384)	<.001	1.176	(0.967;1.385)	<.001	1.175	(0.967;1.384)	<.001	1.177	(0.969;1.386)	<.001
Work status												
Never worked	Ref			Ref			Ref					
Retired	0.311	(0.086;0.537)	0.007	0.249	(−0.323;0.821)	0.393	0.310	(0.084;0.536)	<.01	−0.042	(–0.373;0.289)	0.803
Presently working	0.445	(0.213;0.677)	<.001	0.375	(0.189;0.940)	0.193	0.441	(0.209;0.674)	<.001	0.274	(–0.088;0.635)	0.138
Social participation												
Lesser	Ref			Ref			Ref					
Sometimes	0.330	(0.140;0.520)	<.001	0.329	(0.139;0.519)	<.001	0.511	(0.197;0.825)	<.001	−0.149	(−0.462;0.163)	0.348
More	0.842	(0.633;1.051)	<.001	0.841	(0.632;1.050)	<.001	0.923	(0.631;1.215)	<.001	1.034	(0.585;1.484)	<.001
Depression												
No	Ref			Ref			Ref					
Yes	0.254	(0.003;0.505)	<.05	0.254	(0.003;0.505)	<.05	0.258	(0.007;0.509)	<.01	0.249	(−0.002;0.499)	0.051
WHODAS score												
Low	Ref			Ref			Ref					
Medium	−0.327	(−0.514;-0.141)	<.001	−0.328	(−0.514;-0.141)	<.001	−0.329	(−0.515;-0.142)	<.001	−0.324	(−0.511;-0.138)	<.001
High	−0.937	(−1.139;-0.735)	<.001	−0.939	(−1.142;-0.737)	<.001	−0.934	(−1.136;-0.732)	<.001	−0.936	(−1.138;-0.734)	<.001
Working X gender												
Never work X male				Ref								
Retired X female				0.065	(−0.570;0.700)	0.842						
Presently working X female				0.089	(−0.539;0.717)	0.78						
Social participation X gender												
Lesser X male							Ref					
Sometimes X female							−0.294	(−0.688;0.099)	0.142			
More X female							−0.056	(−0.489;0.377)	0.799			
Work X social participation												
Never work X lesser										Ref		
Retired X sometimes										0.904	(0.440;1.368)	<.001
Retired X more										−0.009	(−0.568;-0.551)	0.976
Presently working X sometimes										0.582	(0.126;1.038)	<.05
Presently working X more										−0.211	(−0.757;0.335)	0.448
Constant	−0.682			−0.615			−0.777			−0.554		
R2	0.378			0.378			0.379			0.381		
Number of observations	5212			5212			5212			5212		
F statistics	166.3			150.4			150.6			138.6		

*Ref* Reference Category

**Fig. 1:**
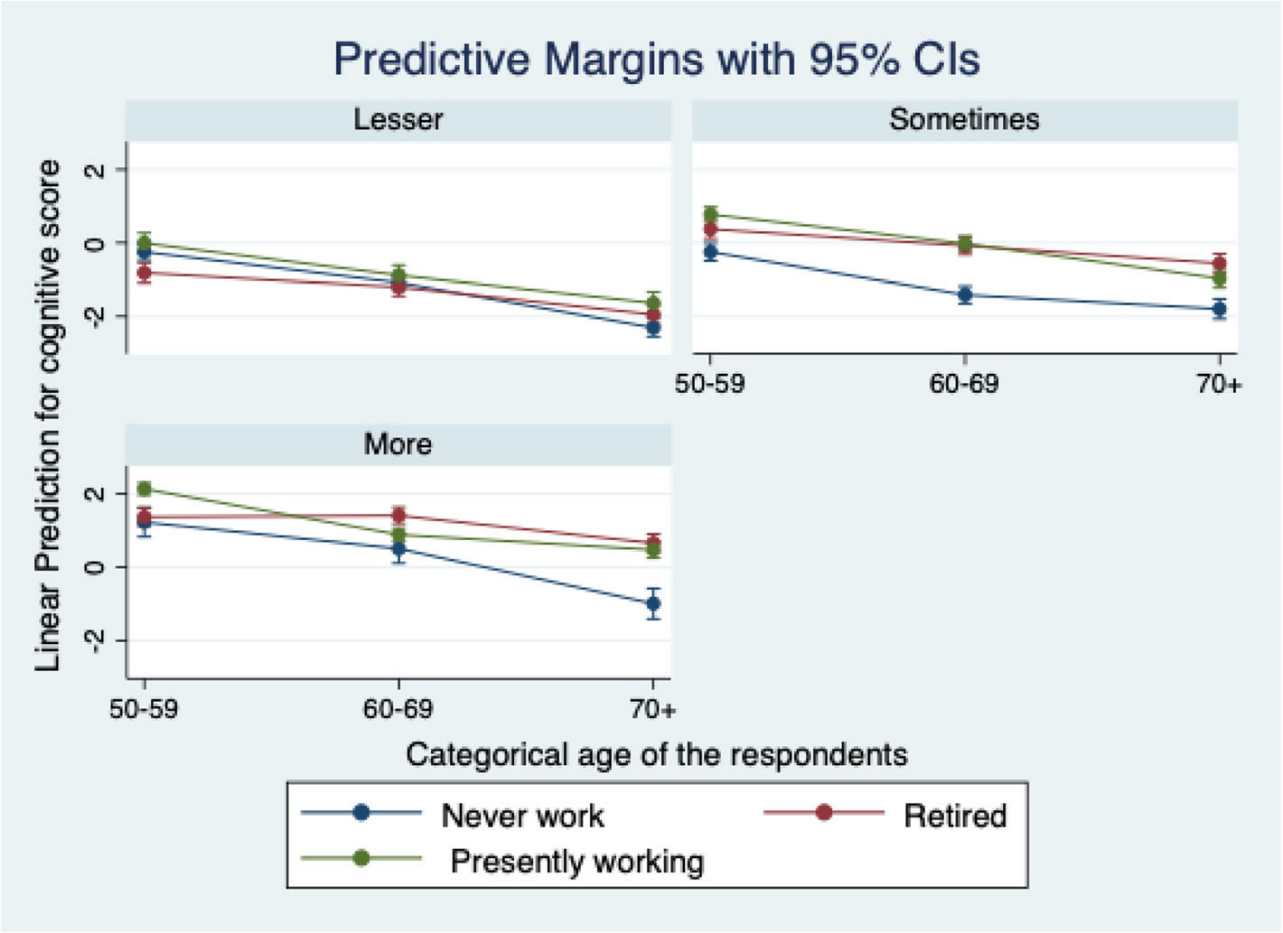
Post estimation of the cognitive score across the level of social participation among elderly

The post-estimation for cognitive scores among the elderly has been illustrated in Fig. 1 with respect to the social participation categories. Higher is the social participation, better is the mean cognitive scores among the elderly in our study. A decline in the cognitive scores was observed with increase in age groups corresponding to 'lesser' social participation both among individuals who had ‘never worked’ and those who had ‘retired’. Individuals who continued to work in the age group of 70 and above had a marginally higher mean cognitive score. Those who participated in social activities ‘sometimes’ and ‘never worked’ had a lower cognitive outcome in every age group compared to those who were ‘presently working’. ‘Retired’ elderly individuals in the 50–59 age group had a lower cognitive score than those ‘presently working’. However, in the age group 60–69 years, ‘retired’ older persons had a matching score with ‘presently working’ older persons. In the category of ‘more’ social participation, elderly who had ‘never worked’ and ‘presently working’ illustrates a declining trend in the mean scores for cognition across the age groups than those are in ‘retired’ category. However, unlike with other social participation categories, those who had already ‘retired’ demonstrated a rise in cognitive scores above ‘presently working’ in the age group of 60–69 years; their scores declined to the level of those ‘presently working’ in the subsequent age group. The elderly who had ‘never worked’ clearly showed a decline in the cognitive scores corresponding to the ‘sometimes’ and ‘more’ categories of social participation across all age groups.

## Discussions

Changes in the life-course activities are stressors of cognitive outcome in later years of life. The rising number of older individuals in India necessitates measurement of one of the under-studied health outcomes, that is, cognition. The study used cross-sectional data to investigate the effect of major participation restrictions on cognitive outcomes of the elderly. Our study made a few important findings, which provide a comprehensive understanding of the impact of changes in the life-course events on cognitive health of the elderly. Elderly who had ‘never worked’ before had the worst mean value for cognitive health compared with the others after adjusting for the selected demographic and socio-economic factors. After controlling the socio-demographic and health factors, it was found that reduction in work and social participation significantly decreased cognitive performance. The pattern of decline was distinctive for the type of social participation practiced by individuals across different levels of labor participation. The theory of disuse perspective explains this nature of activity pattern and cognitive decline of the elderly [18]. The findings are consistent with the studies on retirement that evoke the theory of ‘use it or lose it’. It proves that retirement significantly declines the cognitive health in old age.

Retirement from a highly complicated job negatively affects cognitive abilities in old age. A sudden discontinuation from an intellectually-challenging job makes the deterioration in cognition very pronounced. A long involvement in an occupation, mainly in the informal sectors, stimulates mental abilities up to a certain time. Employment in low-grade jobs and poor educational performance often have a degrading effect on financial security in old age. Lack of pension and social security in the informal labor market compels the elderly to continue to work beyond the statutory age of retirement. Individuals having low levels of education and those belonging to poorer wealth quintiles experience a stressful impact on cognition in old age [31]. In the upcoming decades, more years are expected to be added to the life expectancy of the 60 years and older population. There is a pressing need to address the issue of retirement in regard to the pension system and health expenditure for the elderly population in India [32]. Physical infirmities during old age reduce the working capacity. At the same time, the need to engage in work to overcome financial distress leads to dissatisfaction and depression. The notion of better cognitive functions due to an association with labor market was not supported by our study since financial distress is a reason for continuing work [2].

The effect of retirement on the decline in cognitive outcomes is relatively lower for the age group of 60–69 years. This is substantiated by the theory of *relieved effect* on mental functioning. Retirement from an occupation that is monotonous and repetitive in nature takes away mental worries. Retirement also provides a fair opportunity to engage in long-pending activities having social and cultural value, usually known as the *honeymoon effect* [33]. Other research suggests that retirement and involvement in voluntary activities, meeting with relatives, and participation in other social activities results in cognitive preservation [5]. The initial increase in cognitive outcome in the age group of 60–69 is a good reason to perform socially meaningful activities [14]. The Indian society acknowledges the elderly for their experience in life and their contribution to social activities [22]. For those in the post-retirement phase of life or those who continue to work in older ages, social participation preserves cognitive functions. The interaction between work and social participation shows a positive and significant coefficient with cognition for the selected type in model 4 of multivariate regression. Retirement accompanied with ‘more’ social participation is beneficial for the maintenance of cognitive health in the later years of life as exhibited by Fig. 1. By contrast, ‘more’ social participation by those ‘presently working’ affects their health as they suffer from age-related functional restrictions. Other studies support our findings in that more frequent social participation restricts the cognitive decline regardless of an individual’s level of cognition or physical activity [34]. Therefore, strong social support for the elderly is required to preserve wellbeing in the later stages of life.

Our study didn’t find any gendered pattern for work and social interaction through interactions in the multivariate analysis. This opens the scope for further research to understand the particular patterns for cognitive decline. Exclusion from income-generating activities along with reduced social participation can lead to a long-term deprivation. Such exclusion has a strong impact on mental health, and it also escalates the prevalence of chronic diseases and functional limitations. The evidence of a lower mean score for cognitive scores for depression calls for more social ties to deal with chronic health conditions among the elderly [22].

The present study is a first effort to look into how work status and social participation affect an individual’s mental health. The cross-sectional nature of the data limited the understanding of the long-term effect on cognitive health at different working conditions, as age progresses. We were also limited to include social security measurement to control the effect on cognitive health in old age. However, all of the possible risk factors were taken into consideration.

## Conclusion

Cognitive aging among the elderly is attenuated by an active participation in work and social activities. Retirement from work has a significant impact on the cognitive health of the elderly. Cognitive decline resulting from retirement can be contained if social participation is increased in the later years of life. To control the stressful negative pathways of cognition, it is essential to facilitate the participation of the elderly in social interactions. Cognitive health in later years is a matter of concern as we vigorously discuss healthy aging. This study highlights the pressing need for care and support in the old age to preserve cognitive health. It also makes a case for modifications in the policies and programs on labor participation, with an emphasis on increasing the retirement age to promote healthy aging.

## Additional file

Additional file 1: **Table S1.** Description of the questions and algorithm used for construction of depression in WHO-SAGE (Wave 1) Survey (2008–10). **Table S2.** Descriptive statistics for the cognitive scores of the elderly in India, WHO-SAGE (Wave 1) Survey, 2008–10 (*N* = 5212). **Table S3.** Correlation coefficients and Variance Inflation Factors (VIF) for the co-variates used in the study. (DOCX 128 kb)

## Data Availability

Data is available in the public domain and can be assessed publicly from http://apps.who.int/healthinfo/systems/surveydata/index.php/catalog.

## Abbreviations

ADL: Activities for Daily Living
BWD: Backward Digital Span
FWD: Forward Digital Span
IADL: Instrumental Activities for Daily Living
VF: Verbal Fluency
VR: Verbal Recall
WHO-DAS: World Health Organization Disability Assessment Schedule
WHO-SAGE: World Health Organization Study on global AGEing and adult health
