# Supplementary material for: Impact of transition in work status and social participation on cognitive performance among elderly in India
Source: BMC Geriatr. 2019 Sep 11;19:251. doi: 10.1186/s12877-019-1261-5 (PMC6737668; doi:10.1186/s12877-019-1261-5)
Supplement: Supplementary file 1 — Table S1. Description of the questions and algorithm used for construction of depression in WHO-SAGE (Wave 1) Survey (2008–10). Table S2. Descriptive statistics for the cognitive scores of the elderly in India, WHO-SAGE (Wave 1) Survey, 2008–10 (N = 5212). Table S3. Correlation coefficients and Variance Inflation Factors (VIF) for the co-variates used in the study. (DOCX 128 kb) [file 12877_2019_1261_MOESM1_ESM.docx]

Table 1: Description of the questions and algorithm used for construction of depression in WHO-SAGE (Wave 1) Survey (2008-10).

|  | Sequence | **Questions** |
| --- | --- | --- |
| **Depression** | 1 | During the last 12 months, have you had a period lasting several days when you felt sad, empty or depressed? |
|  | 2 | During the last 12 months, have you had a period lasting several days when you lost interest in most things you usually enjoy such as personal relationships, work or hobbies/recreation? |
|  | 3 | During the last 12 months, have you had a period lasting several days when you have been feeling your energy decreased or that you are tired all the time? |
|  |  | If any of the above three questions are yes then following set of questions were asked |
|  | 4 | Was this period [of sadness/loss of interest/low energy] for more than 2 weeks? |
|  | 5 | Was this period [of sadness/loss of interest/low energy] most of the day, nearly every day? |
|  | 6 | During this period, did you lose your appetite? |
|  | 7 | Did you notice any slowing down in your thinking? |
|  | 8 | Did you notice any problems falling asleep? |
|  | 9 | Did you notice any problems waking up too early? |
|  | 10 | During this period, did you have any difficulties concentrating; for example, listening to others, working, watching TV, listening to the radio? |
|  | 11 | Did you notice any slowing down in your moving around? |
|  | 12 | During this period, did you feel anxious and worried most days? |
|  | 13 | During this period, were you so restless or jittery nearly every day that you paced up and down and couldn’t sit still? |
|  | 14 | During this period, did you feel negative about yourself or like you had lost confidence? |
|  | 15 | Did you frequently feel hopeless - that there was no way to improve things? |
|  | 16 | During this period, did your interest in sex decrease? |
|  | 17 | Did you think of death, or wish you were dead? |
|  | 18 | During this period, did you ever try to end your life? |
|  | **Algorithm** | **To ascertain the depression from this set of questions two set of variables were computed. First set was based on the questions1, 2, 3, 4, 5 and 16. From this set three variables were computed taking values 0 and 1: a) first variable takes value 1 if response to any of questions 1, 4, and 5 was yes. b) second variable takes value 1 if question 2 or 16 has response yes. c) the third variable takes value 1 if question 3 has response yes. The second set of variables was based on questions 6, 7, 8, 9, 10, 11, 12, 13, 14, 15, 17 and 18. From these questions seven variables were computed. a)first variable takes value 1 if response to questions 14 or 15 is yes. b) second variable takes value 1 if response to questions 12 or 13 is yes. c) third variable takes value 1 if questions 17 or 18 has response yes. d) fourth variable takes value 1 if questions 7 or 10 has response yes. e) fifth variable takes value 1 if response to questions 11 is yes. f) sixth variable takes value 1 if response to questions 8 or 9 is yes. g) seventh variable takes value 1 if the response to question6 is yes. These newly created variable from the respective sets were added to obtain two new variables : first consisting sum of first set of variables (maximum value 3) and second consisting sum of second set of variables (maximum value 7). Based on these two variables, a respondent is said to suffer from depression if he has value for the first variable to be 2-plus and the value for second variable to be 4-plus.** |

Reference for Table 1: Arokiasamy P, Uttamacharya U, Jain K, Biritwum RB, Yawson AE, Wu F, et al. The impact of multimorbidity on adult physical and mental health in low- and middle-income countries : what does the study on global ageing and adult health ( SAGE ) reveal ? BMC Med. 2015;13:1–16. doi:10.1186/s12916-015-0402-8.

Table 2: Descriptive statistics for the cognitive scores of the elderly in India, WHO-SAGE (Wave 1) Survey, 2008-10 (N=5212)

| **Categories** | **Percentage** | **Mean** | **SD** | **Minimum** | **Maximum** | **Median** | **Interquartile range** |
| --- | --- | --- | --- | --- | --- | --- | --- |
| **Age Groups** |  |  |  |  |  |  |  |
| 50-59 | 47.39 | 0.79 | 3.40 | -9.86 | 12.80 | 0.73 | -1.51, 3.13 |
| 60-69 | 33.71 | 0.00 | 3.33 | -10.20 | 12.52 | -0.09 | -2.34, 2.32 |
| 70+ | 18.90 | -1.09 | 3.43 | -9.59 | 13.46 | -1.21 | -3.45, 1.20 |
| **Sex** |  |  |  |  |  |  |  |
| Male | 53.74 | 1.04 | 3.32 | -9.41 | 13.46 | 1.07 | -1.16, 3.32 |
| Female | 46.26 | -0.84 | 3.33 | -10.20 | 11.76 | -1.03 | -3.14, 1.30 |
| **Place of residence** | |  |  |  |  |  |  |
| Urban | 26.19 | 1.37 | 3.47 | -9.86 | 12.39 | 1.37 | -0.88, 3.69 |
| Rural | 73.81 | -0.25 | 3.35 | -10.20 | 13.46 | -0.34 | -2.64, 2.02 |
| **Marital Status** |  |  |  |  |  |  |  |
| Never Married | 0.94 | 1.26 | 4.13 | -6.37 | 11.72 | 1.40 | -2.69, 4.04 |
| Currently Married | 76.82 | 0.59 | 3.36 | -10.20 | 13.46 | 0.54 | -1.72, 2.91 |
| Others | 22.24 | -1.33 | 3.31 | -9.86 | 10.37 | -1.43 | -3.69, 0.82 |
| **Caste** |  |  |  |  |  |  |  |
| Unreserved | 62.24 | 0.54 | 3.48 | -9.86 | 13.46 | 0.54 | -1.86, 2.93 |
| Reserved | 37.76 | -0.43 | 3.33 | -10.20 | 12.52 | -0.53 | -2.77, 1.71 |
| **Completed education** | |  |  |  |  |  |  |
| No formal | 48.16 | -1.53 | 2.91 | -10.20 | 11.00 | -1.58 | -3.56, 0.39 |
| Primary | 25.92 | 0.63 | 2.82 | -9.31 | 10.19 | 0.60 | -1.23, 2.65 |
| Secondary | 20.40 | 2.52 | 3.00 | -8.83 | 13.46 | 2.58 | 0.54, 4.46 |
| College | 5.53 | 4.20 | 2.91 | -8.83 | 12.39 | 4.20 | 2.31, 5.99 |
| **Wealth Index** |  |  |  |  |  |  |  |
| Low |  | -1.24 | 3.07 | -10.20 | 10.92 | -1.29 | -3.39, 0.83 |
| Medium |  | 0.05 | 3.27 | -9.86 | 11.56 | 0.00 | -2.23, 2.24 |
| High |  | 1.59 | 3.41 | -8.83 | 13.46 | 1.55 | -0.72, 3.89 |
| **Work Status** |  |  |  |  |  |  |  |
| Never worked | 26.38 | -0.74 | 3.34 | -10.20 | 11.00 | -0.98 | -2.99, 1.47 |
| Retired | 29.45 | -0.03 | 3.59 | -9.59 | 13.46 | -0.03 | -2.64, 2.43 |
| Presently working | 44.17 | 0.85 | 3.28 | -9.86 | 12.80 | 0.73 | -1.40, 3.11 |
| **Social Participation** | |  |  |  |  |  |  |
| Lesser |  | -0.99 | 3.28 | -9.86 | 11.00 | -1.10 | -3.26, 1.23 |
| Sometimes |  | -0.14 | 3.31 | -10.20 | 11.18 | -0.20 | -2.55, 2.24 |
| More |  | 1.41 | 3.32 | -8.27 | 13.46 | 1.35 | -0.85, 3.66 |
| **Depression** |  |  |  |  |  |  |  |
| No | 89.72 | 0.25 | 3.47 | -9.86 | 13.46 | 0.21 | -2.13, 2.63 |
| Yes | 10.28 | -0.53 | 3.26 | -10.20 | 10.93 | -0.42 | -2.78, 1.68 |
| **WHODAS score** | |  |  |  |  |  |  |
| Low |  | 1.41 | 3.42 | -9.86 | 13.46 | 1.42 | -0.77, 3.64 |
| Medium |  | 0.11 | 3.14 | -9.03 | 10.50 | 0.06 | -2.04, 2.32 |
| High |  | -1.15 | 3.29 | -10.20 | 11.00 | -1.23 | -3.41, 0.99 |
| **Total** |  | 0.17 | 3.45 | -10.20 | 13.46 | 0.12 | -2.21, 2.56 |

Table 3:Correlation coefficients and Variance Inflation Factors (VIF) for the co-variates used in the study

|  | **Cognition Score** | **Age** | **Sex** | **Place of residence** | **Caste** | **WHODAS score** | **Education** | **Marital status** | **Wealth Index** | **Social Participation** | **Depression** | **VIF** |
| --- | --- | --- | --- | --- | --- | --- | --- | --- | --- | --- | --- | --- |
| **Cognition Score** | 1 |  |  |  |  |  |  |  |  |  |  |  |
| **Age** | -0.20 | 1.00 |  |  |  |  |  |  |  |  |  | 1.2 |
| **Sex** | -0.27 | -0.07 | 1.00 |  |  |  |  |  |  |  |  | 1.56 |
| **Place of residence** | -0.21 | 0.00 | -0.05 | 1.00 |  |  |  |  |  |  |  | 1.17 |
| **Caste** | -0.14 | -0.02 | 0.00 | 0.03 | 1.00 |  |  |  |  |  |  | 1.04 |
| **WHODAS score** | -0.30 | 0.26 | 0.22 | 0.08 | 0.06 | 1.00 |  |  |  |  |  | 1.23 |
| **Education** | 0.54 | -0.11 | -0.38 | -0.28 | -0.11 | -0.27 | 1.00 |  |  |  |  | 1.58 |
| **Marital status** | -0.23 | 0.25 | 0.30 | -0.01 | 0.04 | 0.18 | -0.22 | 1.00 |  |  |  | 1.22 |
| **Wealth Index** | 0.33 | -0.03 | -0.01 | -0.29 | -0.14 | -0.13 | 0.39 | -0.08 | 1.00 |  |  | 1.29 |
| **Social Participation** | 0.28 | -0.10 | -0.42 | 0.02 | 0.03 | -0.19 | 0.28 | -0.20 | 0.11 | 1.00 |  | 1.27 |
| **Depression** | -0.07 | 0.05 | 0.04 | 0.05 | 0.00 | 0.20 | -0.10 | 0.04 | -0.11 | -0.03 | 1.00 | 1.05 |
